# Supplementary material for: High Structural Diversity of Aeruginosins in Bloom-Forming Cyanobacteria of the Genus Planktothrix as a Consequence of Multiple Recombination Events
Source: Mar Drugs. 2023 Dec 13;21(12):638. doi: 10.3390/md21120638 (PMC10744761; doi:10.3390/md21120638)
Supplement: Supplementary file 1 [file marinedrugs-21-00638-s001.zip › Suppl_Figures_13_12_23.pdf]

Supplementary Information

High structural diversity of aeruginosins in bloom-forming cyanobacteria of the genus *Planktothrix* as a consequence of multiple recombination events

Elisabeth Entfellner[1,2], Kathrin B.L. Baumann[1#], Christine Edwards[3], Rainer Kurmayer[1,2*]

[1] Research Department for Limnology, Universität Innsbruck, Mondseestrasse 9, 5310 Mondsee, Austria, elisabeth.entfellner@gmx.at; katbaumann87@gmail.com

[2] Universität Innsbruck, Innrain 52, 6020 Innsbruck, Austria;

[3] CyanoSol Research Group, Pharmacy & Life Sciences, Robert Gordon University, Aberdeen, AB10 7GJ UK c.edwards@rgu.ac.uk

*Corresponding Author: rainer.kurmayer@uibk.ac.at

[#] Current address: Department of Surface Waters-Research and Management, Swiss Federal Institute for Aquatic Science and Technology (EAWAG), 6047 Kastanienbaum, Switzerland.

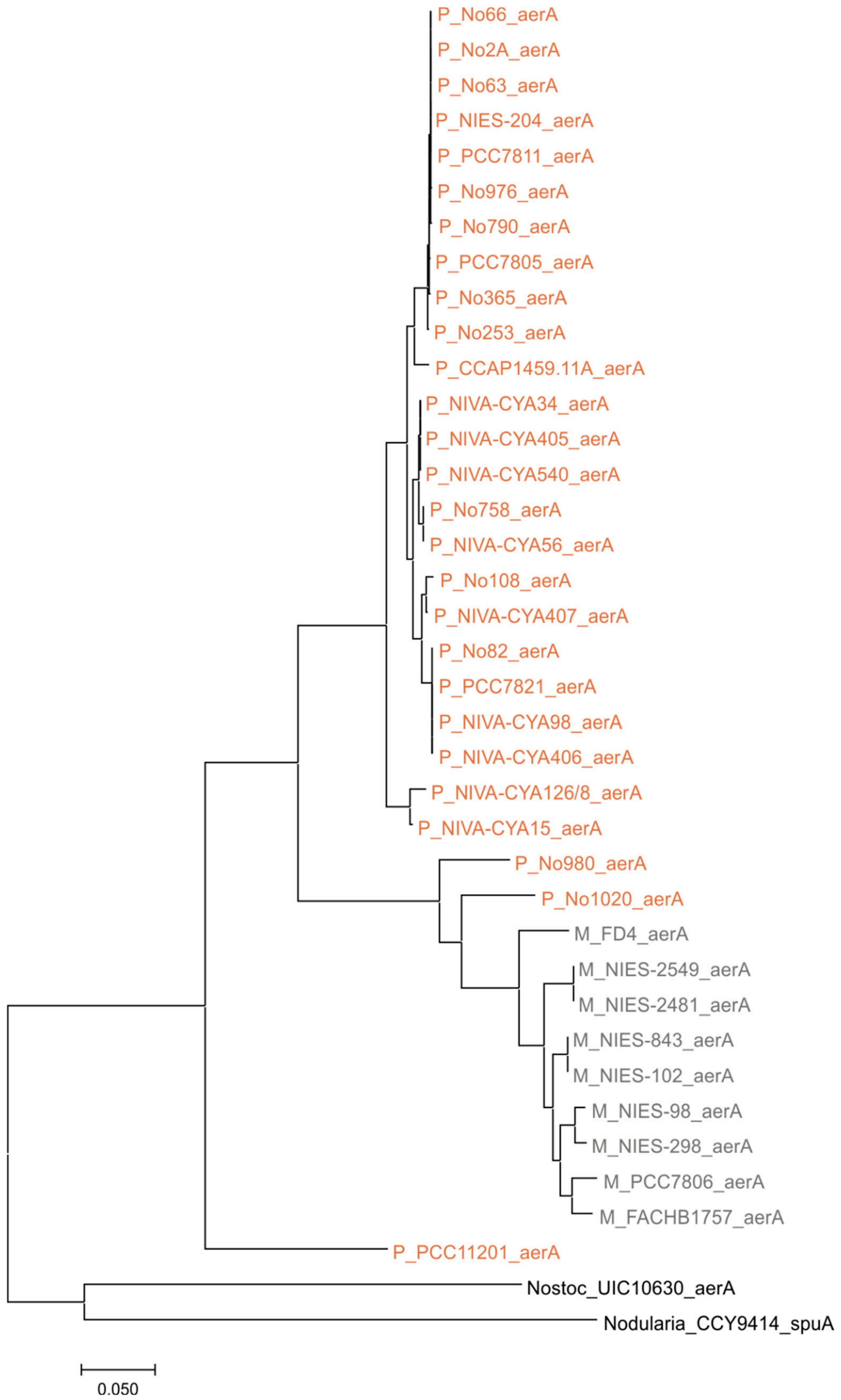

Figure S1. Maximum likelihood phylogenetic tree based on *aerA* (nucleotide) sequence (alignment: 4407 bp). P_, M_ indicate genera *Planktothrix* or *Microcystis*, each. *Nostoc* UIC10630 and *Nodularia* CCY9414 served as outgroups.

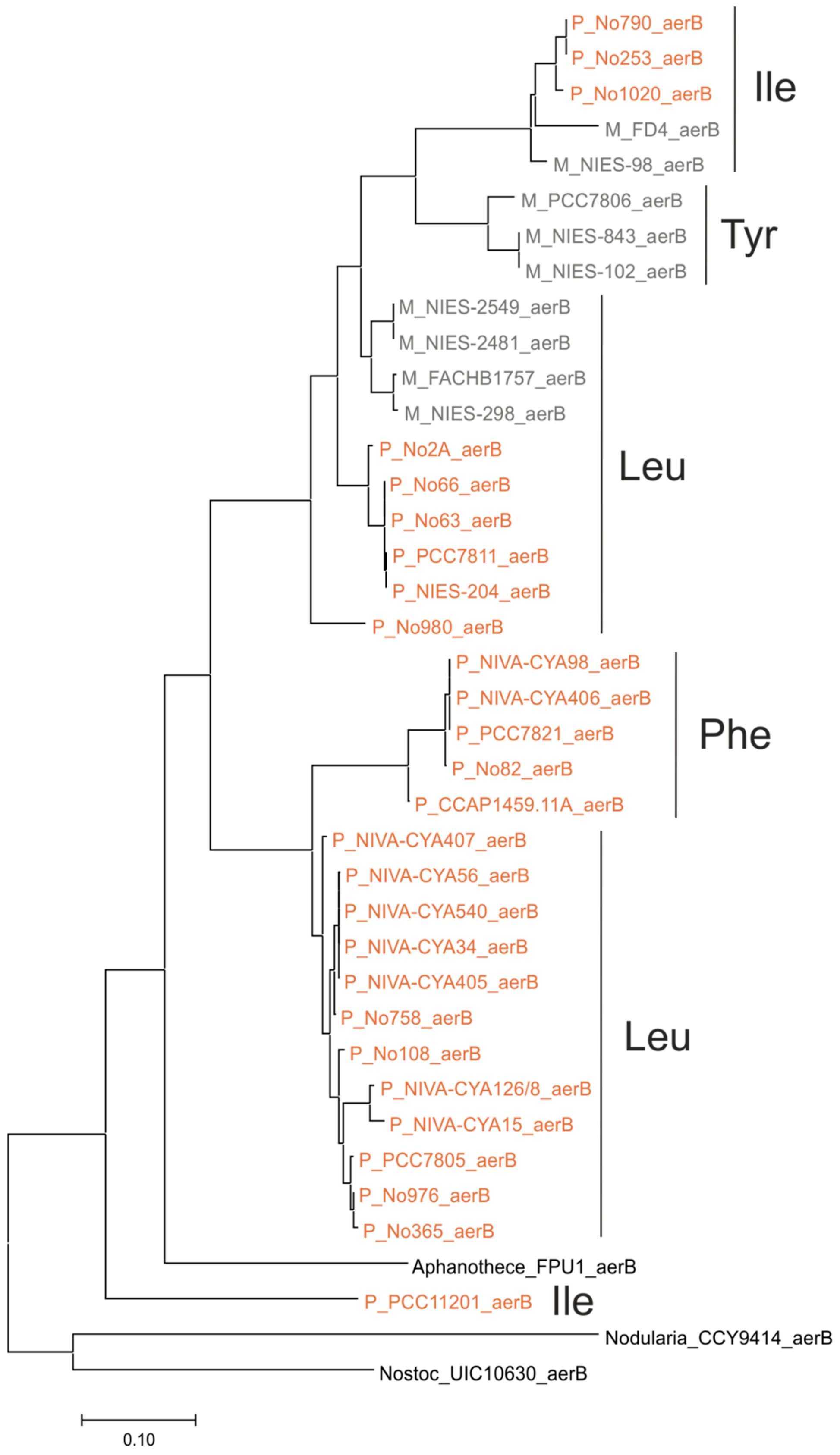

Figure S2. Maximum likelihood phylogenetic tree based on *aerB* (nucleotide) sequence (alignment: 4857 bp). P_, M_ indicate genera *Planktothrix* or *Microcystis*, each. *Nostoc* UIC10630 and *Nodularia* CCY9414 served as outgroups. Using the NRPS prediction tool [26] four different specificity-conferring codes for the adenylation domain of AerB were identified: Leu, Ile, Phe and Tyr.

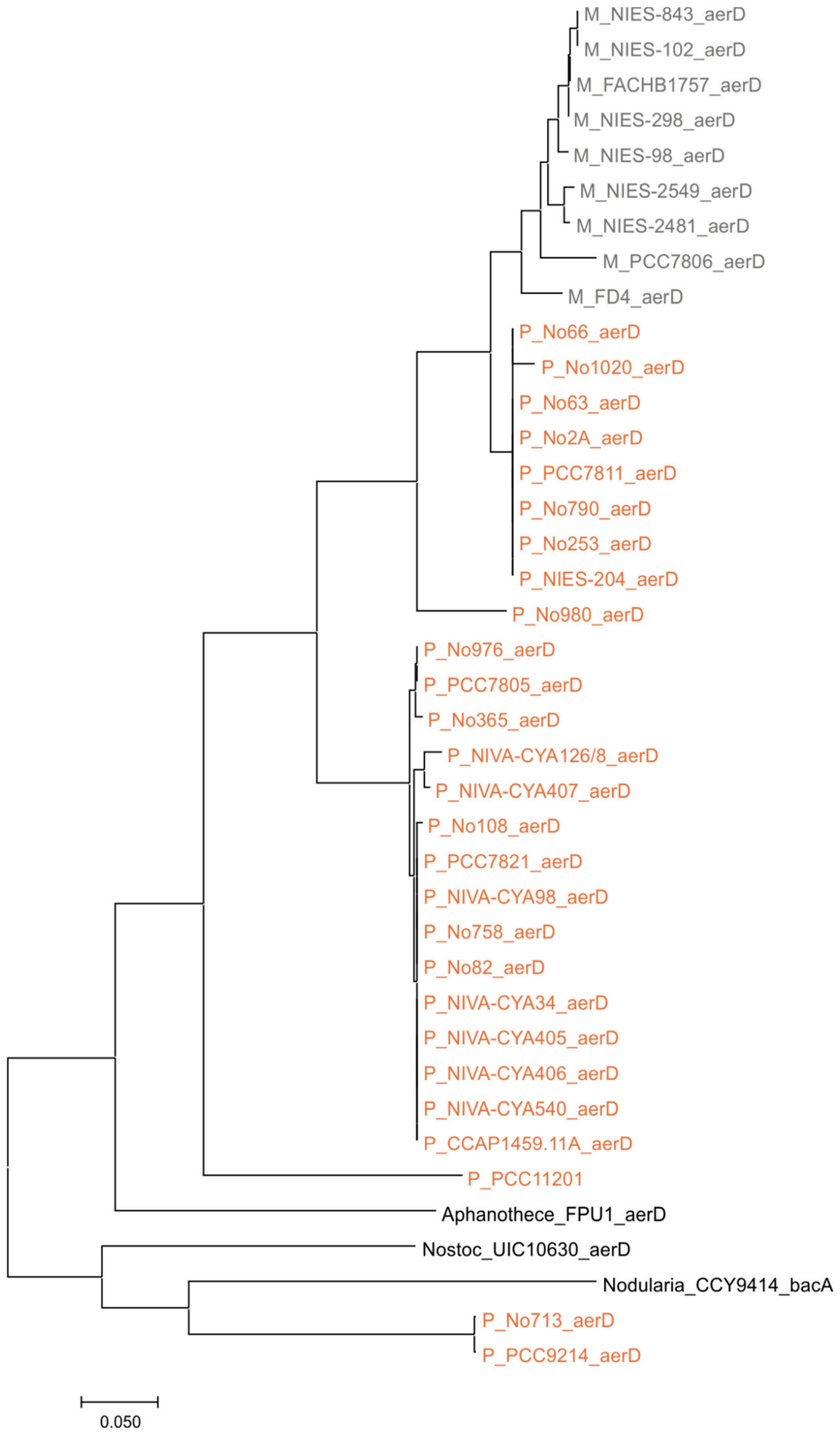

Figure S3. Maximum likelihood phylogenetic tree based on *aerD* (nucleotide) sequence (alignment: 633 bp). P_, M_ indicate genera *Planktothrix* or *Microcystis*, each. *Nostoc* UIC10630 and *Nodularia* CCY9414 served as outgroups.

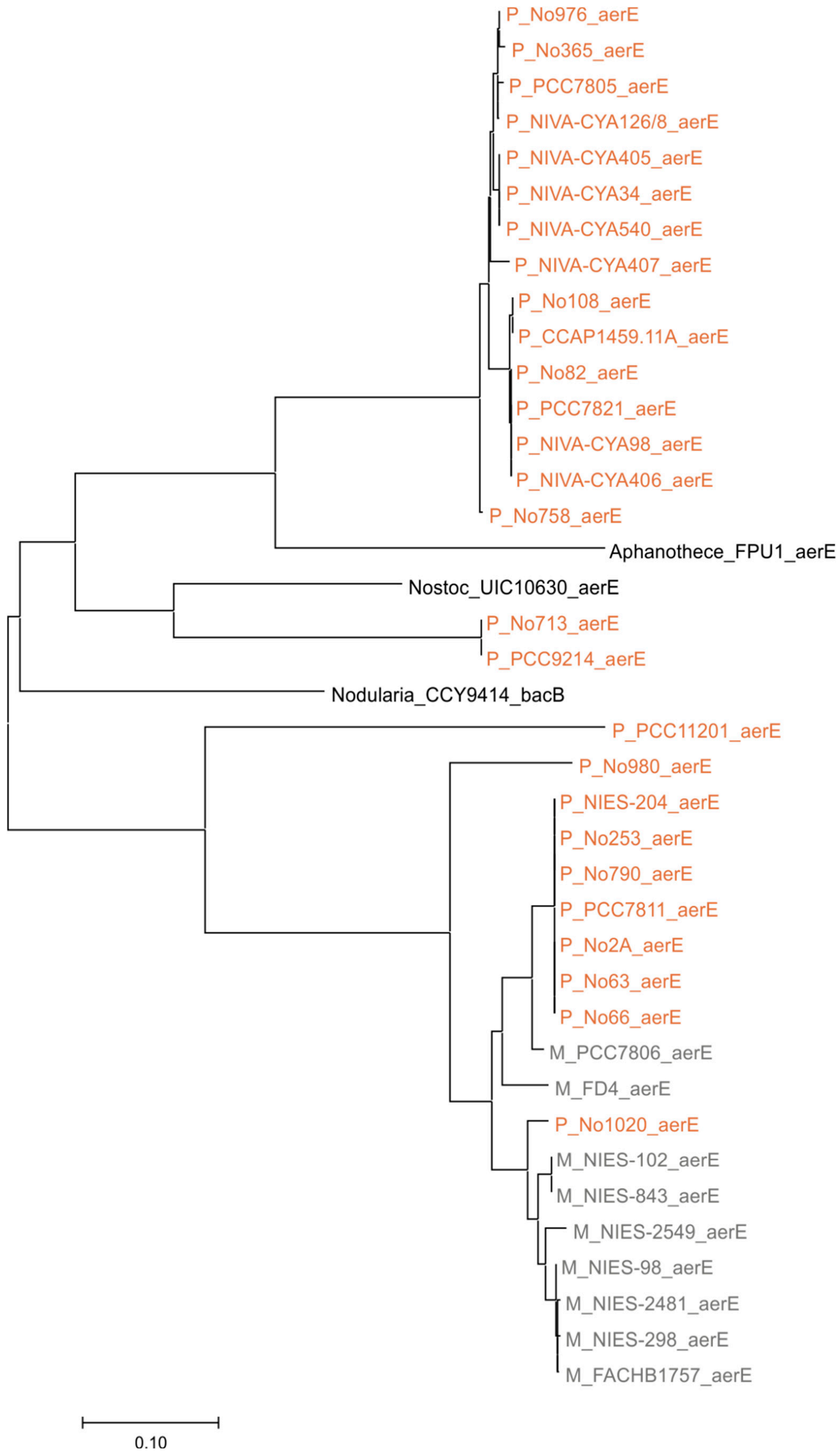

Figure S4. Maximum likelihood phylogenetic tree based on *aerE* (nucleotide) sequence (alignment: 756 bp). P_, M_ indicate genera *Planktothrix* or *Microcystis*, each. *Nostoc* UIC10630 and *Nodularia* CCY9414 served as outgroups.

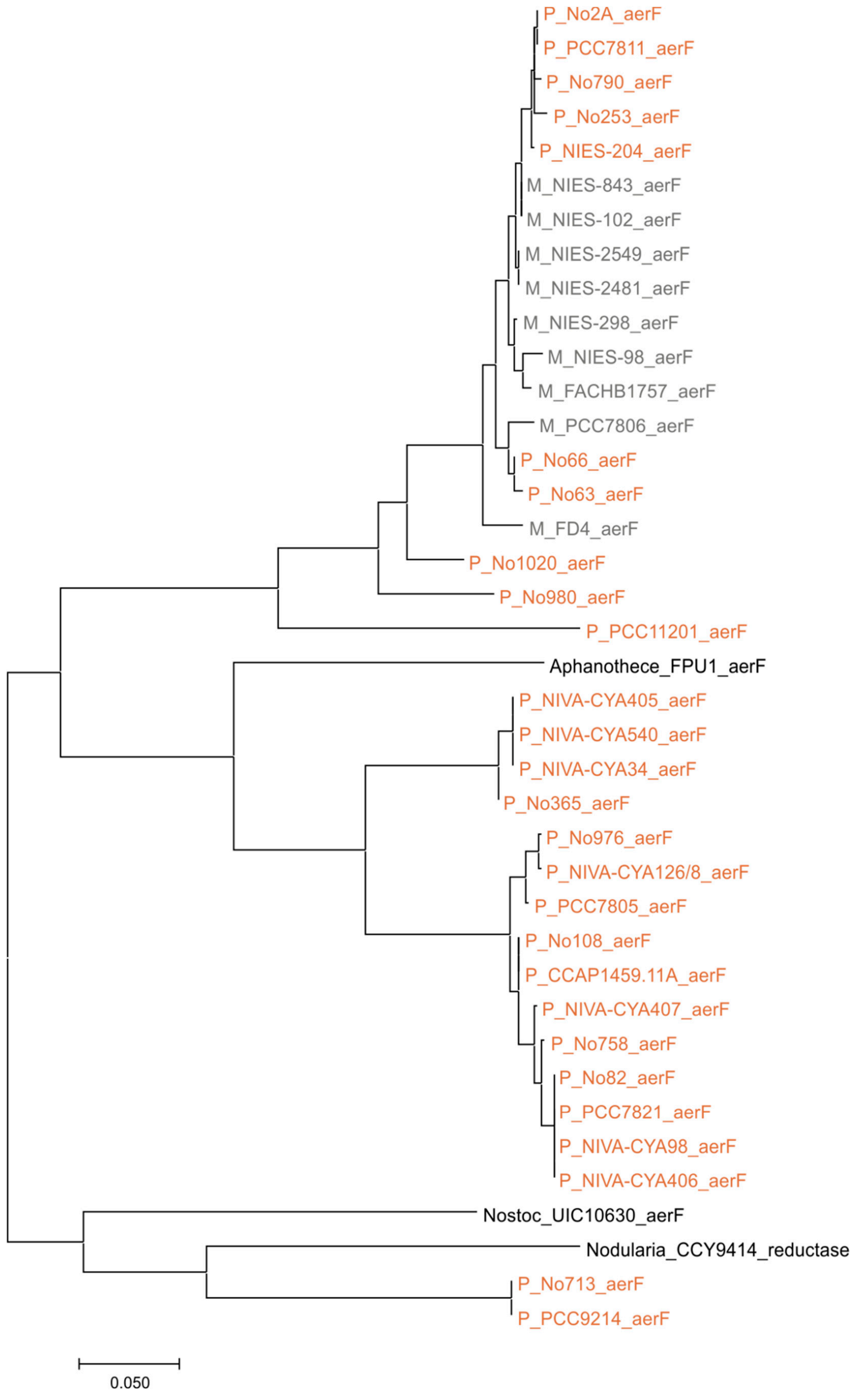

Figure S5. Maximum likelihood phylogenetic tree based on *aerF* (nucleotide) sequence (alignment: 801 bp). P_, M_ indicate genera *Planktothrix* or *Microcystis*, each. *Nostoc* UIC10630 and *Nodularia* CCY9414 served as outgroups.

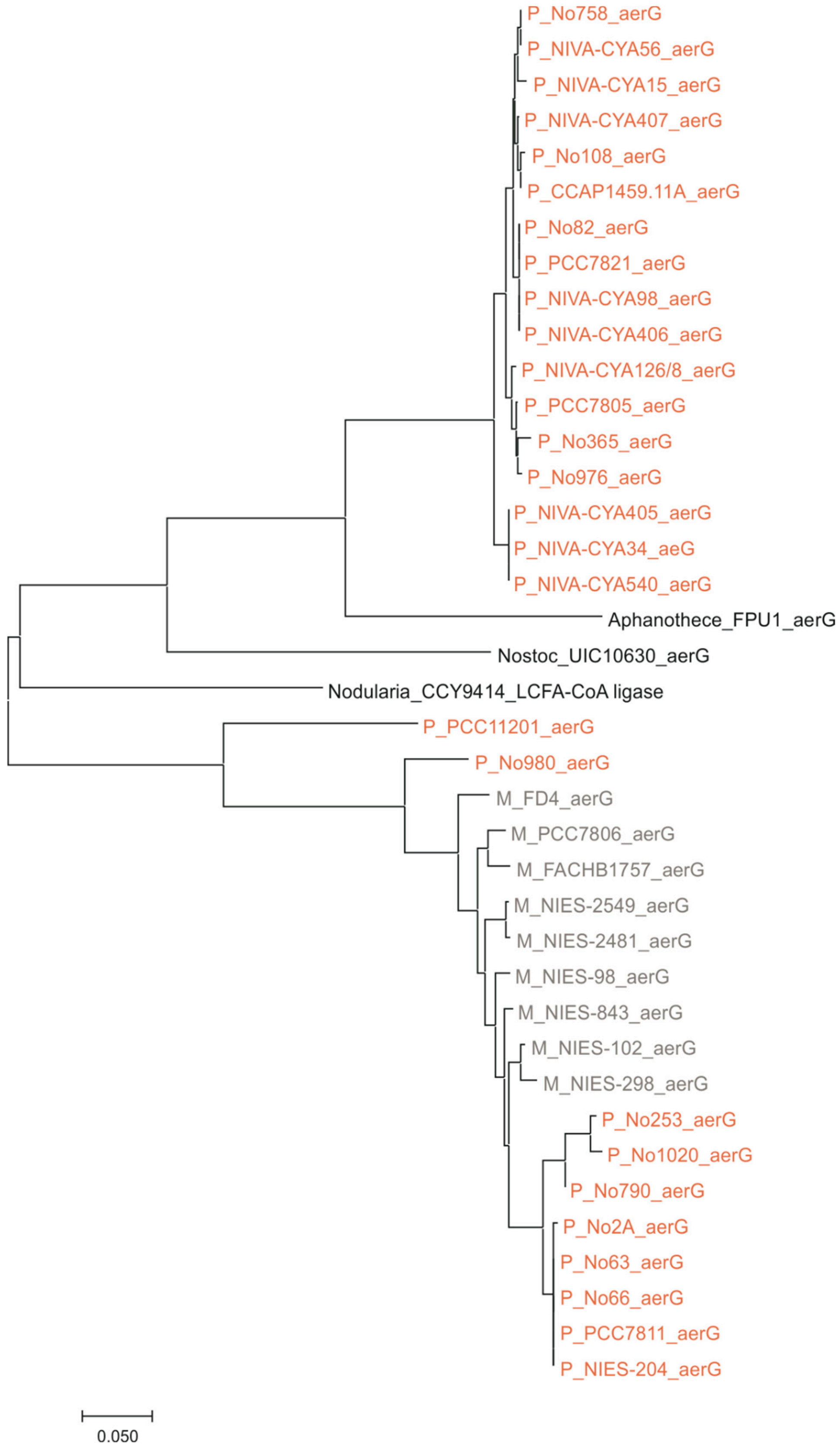

Figure S6. Maximum likelihood phylogenetic tree based on *aerG* (nucleotide) sequence (alignment: 3366 bp). P_, M_ indicate genera *Planktothrix* or *Microcystis*, each. *Nostoc* UIC10630 and *Nodularia* CCY9414 served as outgroups.

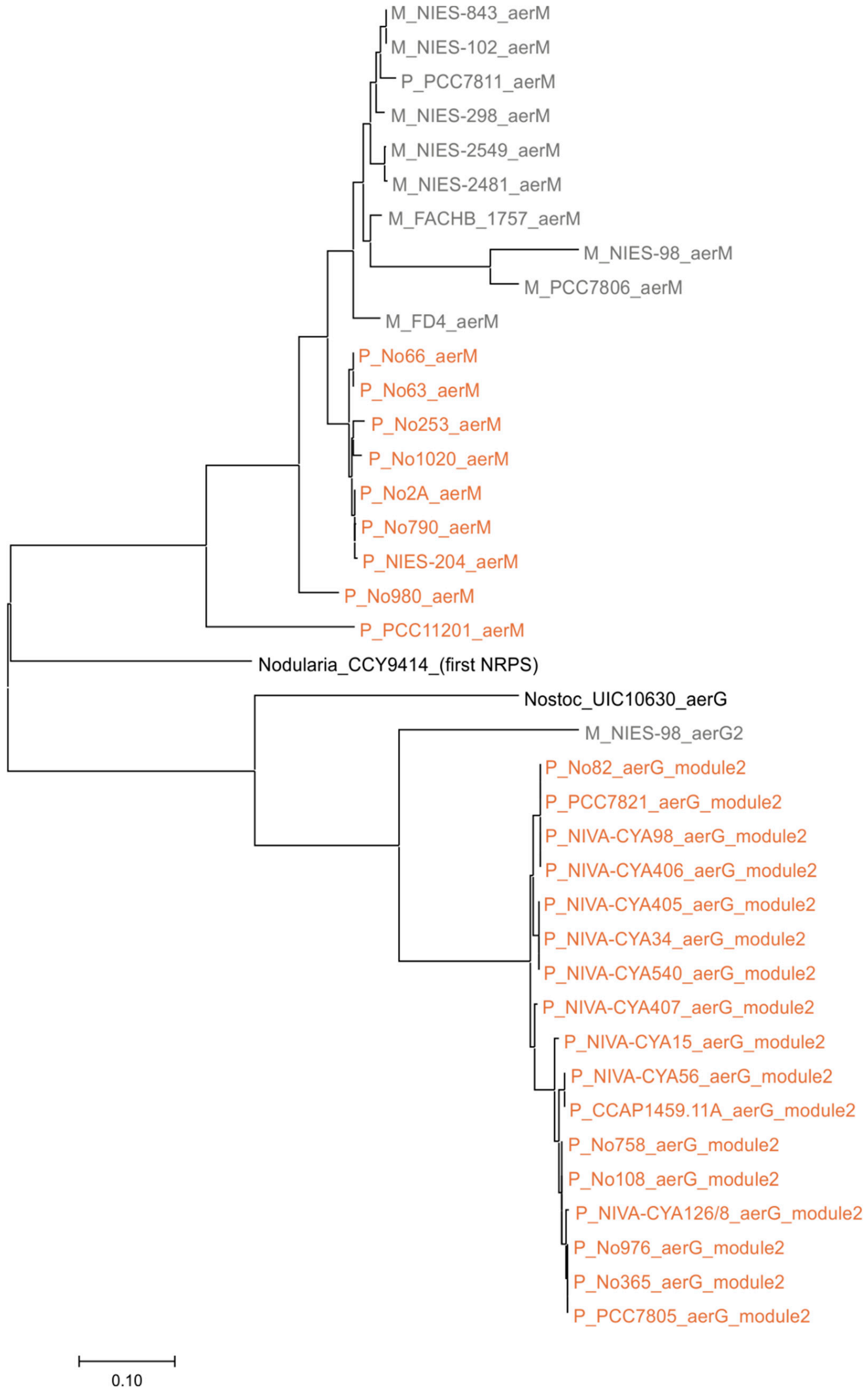

Figure S7. Maximum likelihood phylogenetic tree based on *aerG*_module2 and *aerM* (nucleotide) sequence (alignment: 4533 bp). P_, M_ indicate genera *Planktothrix* or *Microcystis*, each. *Nostoc* UIC10630 and *Nodularia* CCY9414 served as outgroups.

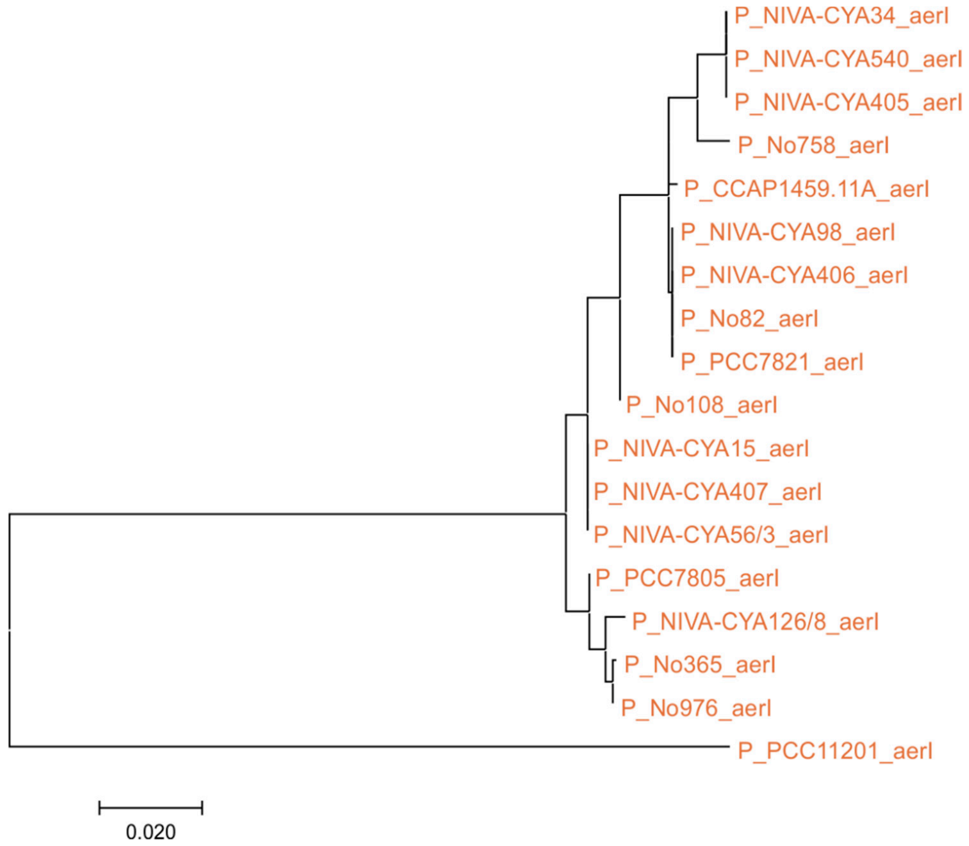

Figure S8. Maximum likelihood phylogenetic tree based on *aerI* (nucleotide) sequence (alignment: 1260 bp). P_, M_ indicate genera *Planktothrix* or *Microcystis*, each.

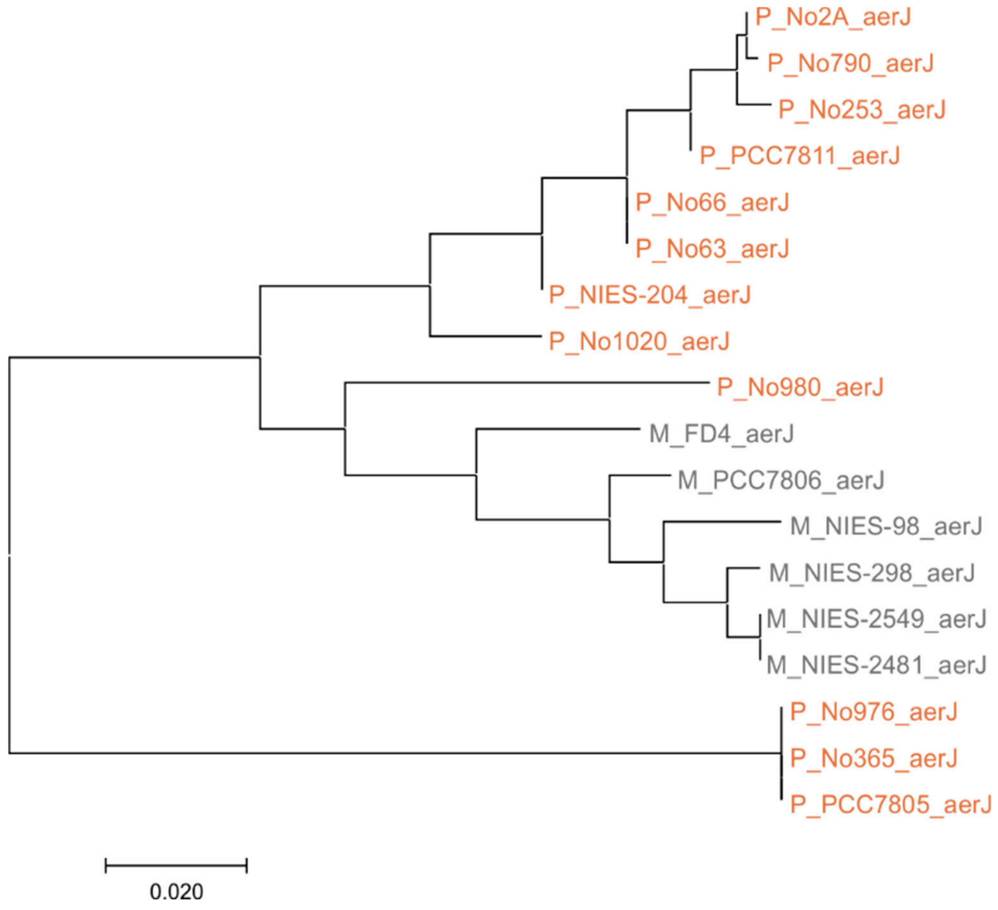

Figure S9. Maximum likelihood phylogenetic tree based on *aerJ* (nucleotide) sequence (alignment: 1905 bp). P_, M_ indicate genera *Planktothrix* or *Microcystis*, each.

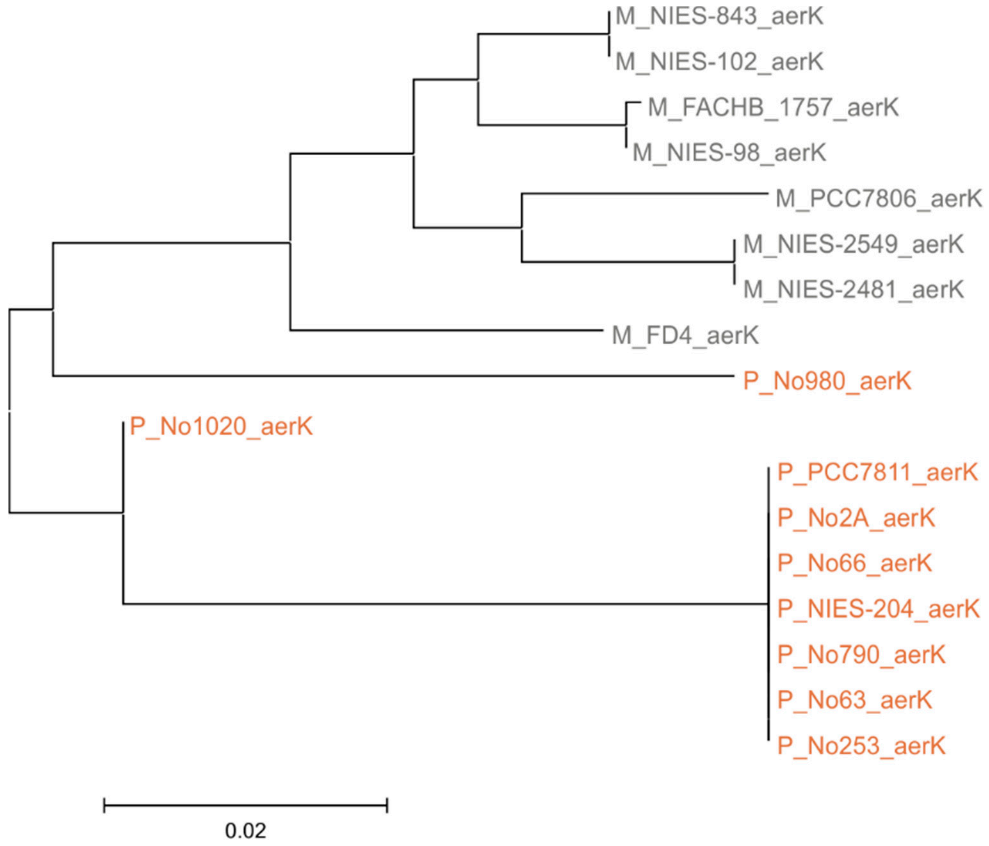

Figure S10. Maximum likelihood phylogenetic tree based on *aerK* (nucleotide) sequence (alignment: 1044 bp). P_, M_ indicate genera *Planktothrix* or *Microcystis*, each.

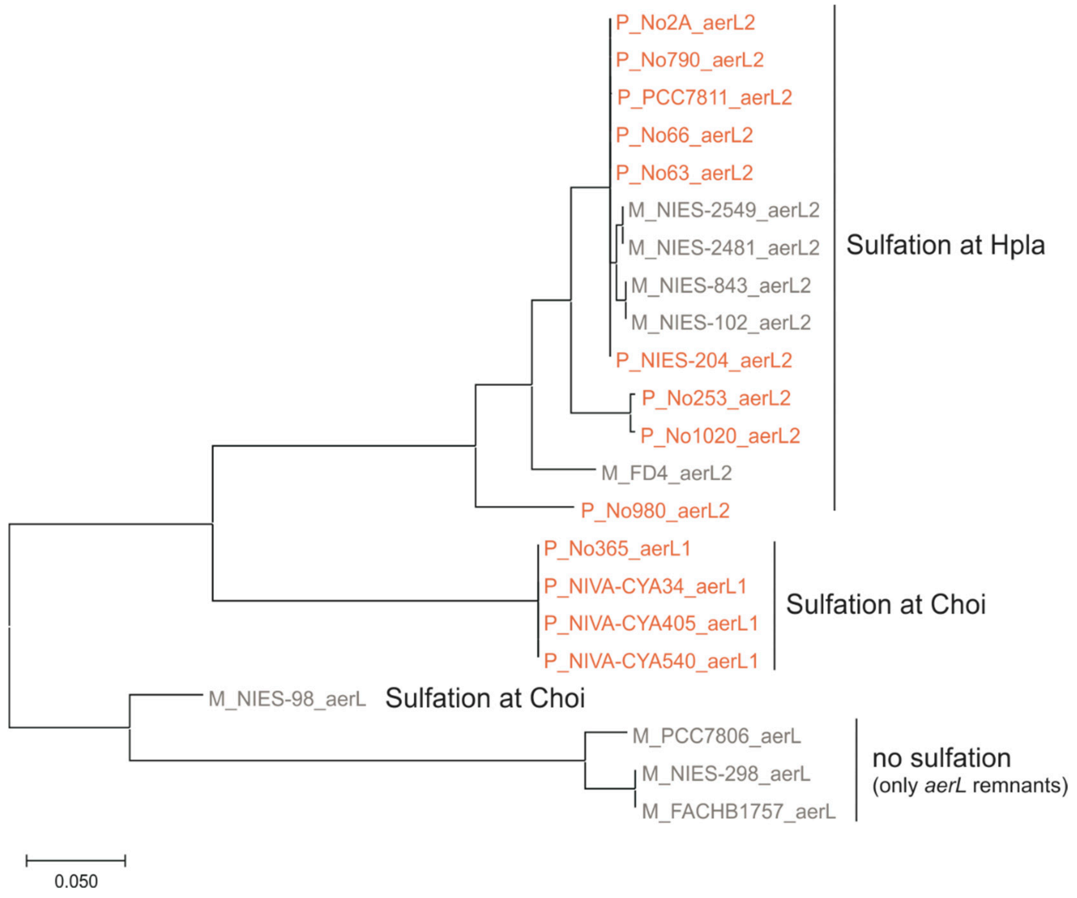

Figure S11. Maximum likelihood phylogenetic tree based on *aerL* (nucleotide) sequence (alignment: 1011 bp). P_, M_ indicate genera *Planktothrix* or *Microcystis*, each. In addition the putative sulfation reactions are indicated (Figure 4).

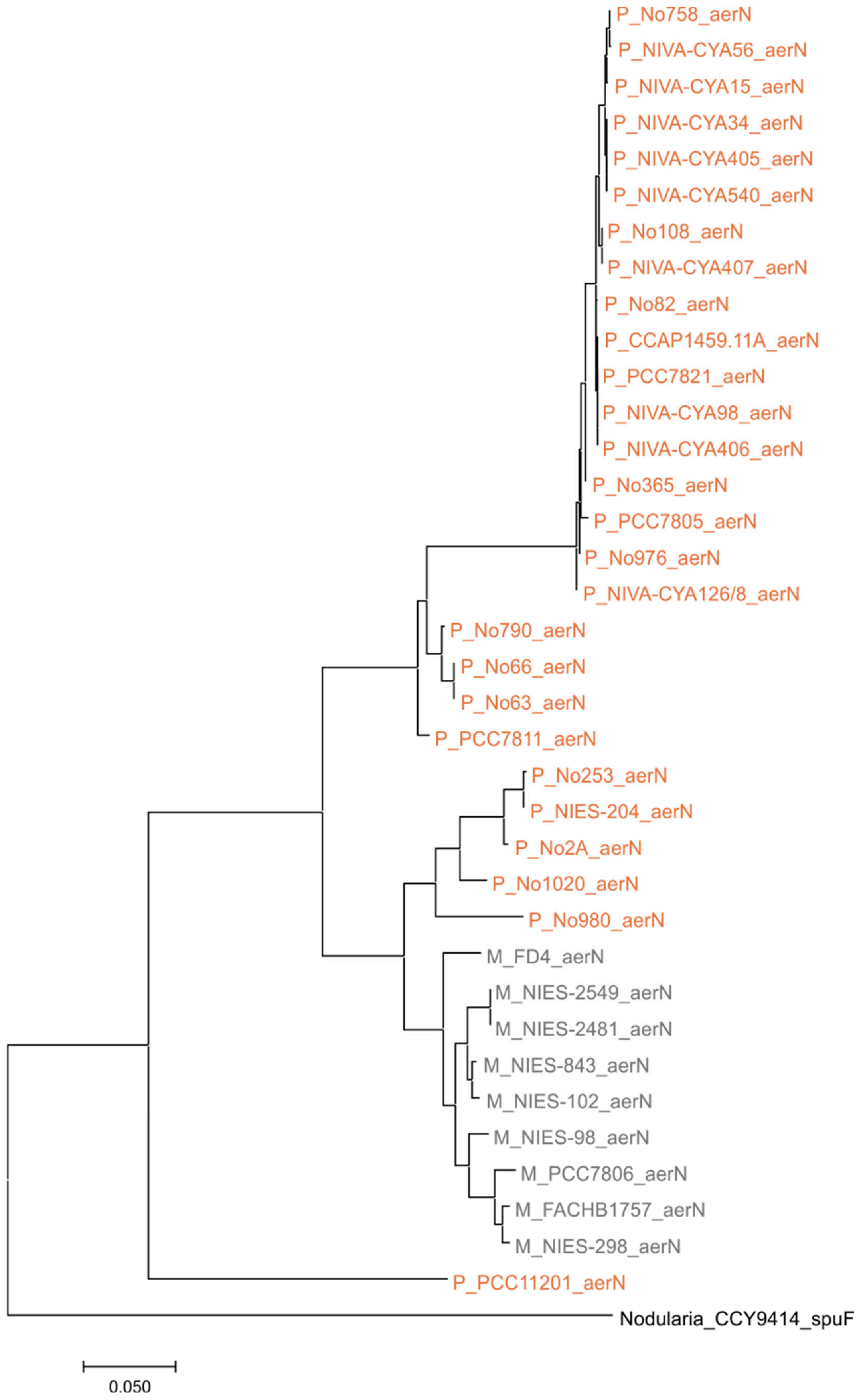

Figure S12. Maximum likelihood phylogenetic tree based on *aerN* (nucleotide) sequence (alignment: 2058 bp). P_, M_ indicate genera *Planktothrix* or *Microcystis*, each. *Nodularia* CCY9414 served as outgroup.

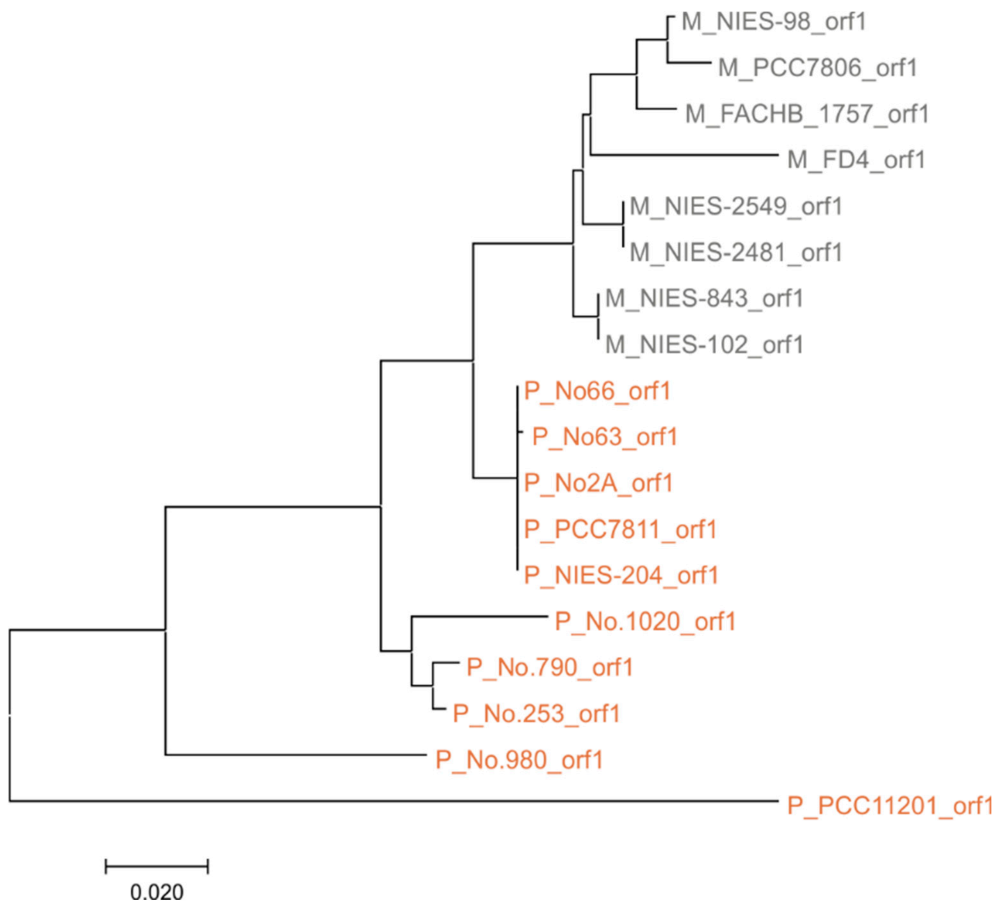

Figure S13. Maximum likelihood phylogenetic tree based on ORF1 (nucleotide) sequence (alignment: 786 bp). P_, M_ indicate genera *Planktothrix* or *Microcystis*, each.

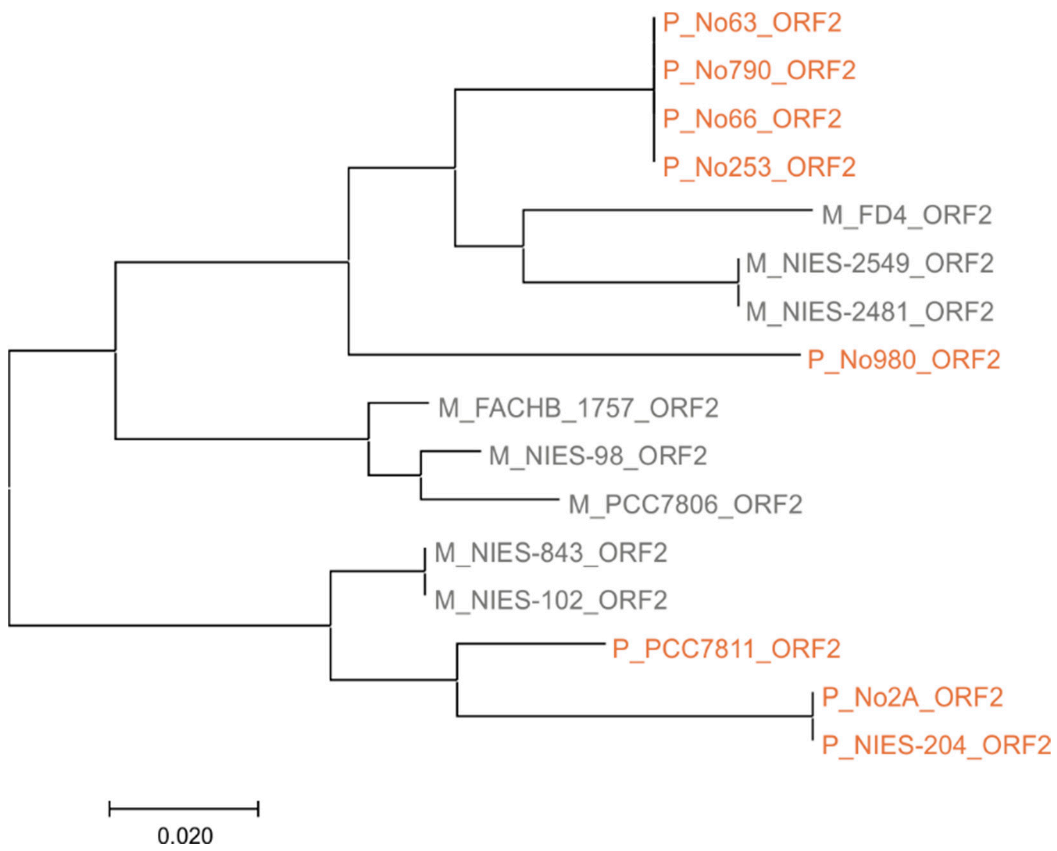

Figure S14. Maximum likelihood phylogenetic tree based on ORF2 (nucleotide) sequence (alignment: 627 bp). P_, M_ indicate genera *Planktothrix* or *Microcystis*, each.

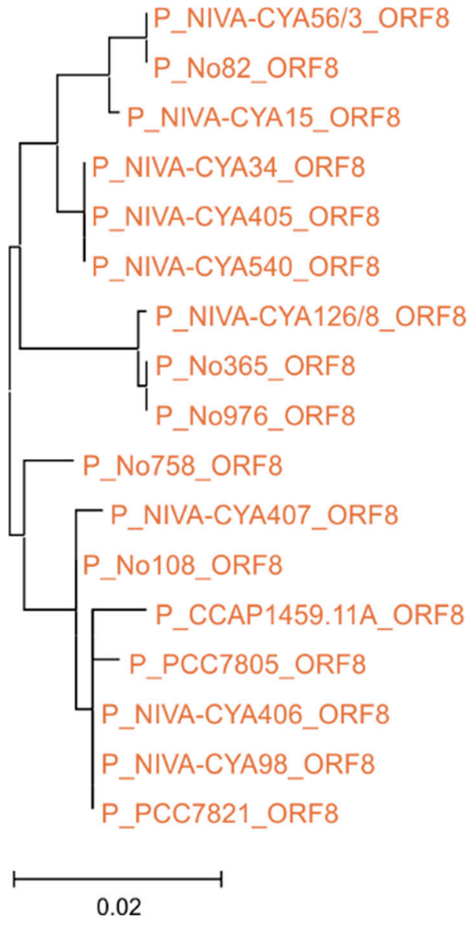

Figure S15. Maximum likelihood phylogenetic tree based on ORF8 (nucleotide) sequence (alignment: 1161 bp). P_ indicates genus *Planktothrix*.

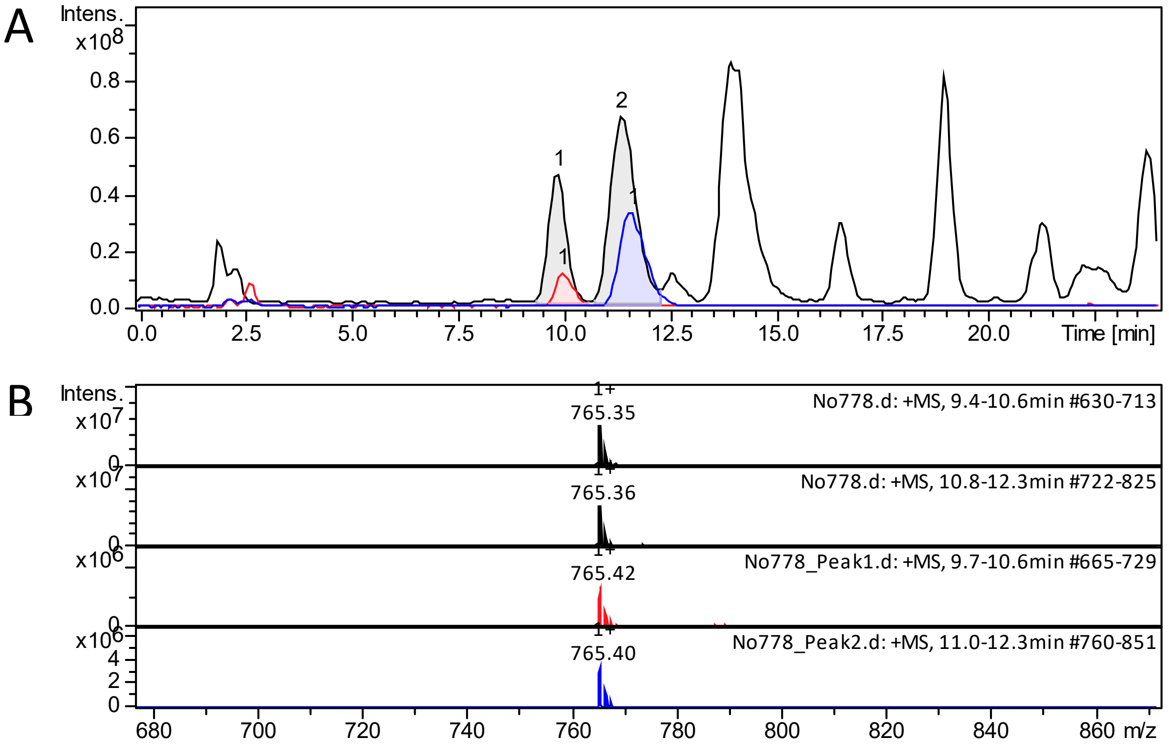

Figure S16. (A) Base peak chromatograms (BPC) of methanolic peptide extracts from *Planktothrix* strain No778 with first injection (black), re-injection of peak 1 (red) and re-injection of peak 2 (blue). **(B)** Mass peak chromatograms [m z^{-1}] of peak 1 and 2 from the first injection (black), re-injection of peak 1 (red) and re-injection of peak 2 (blue); [m z^{-1}] 765.3.

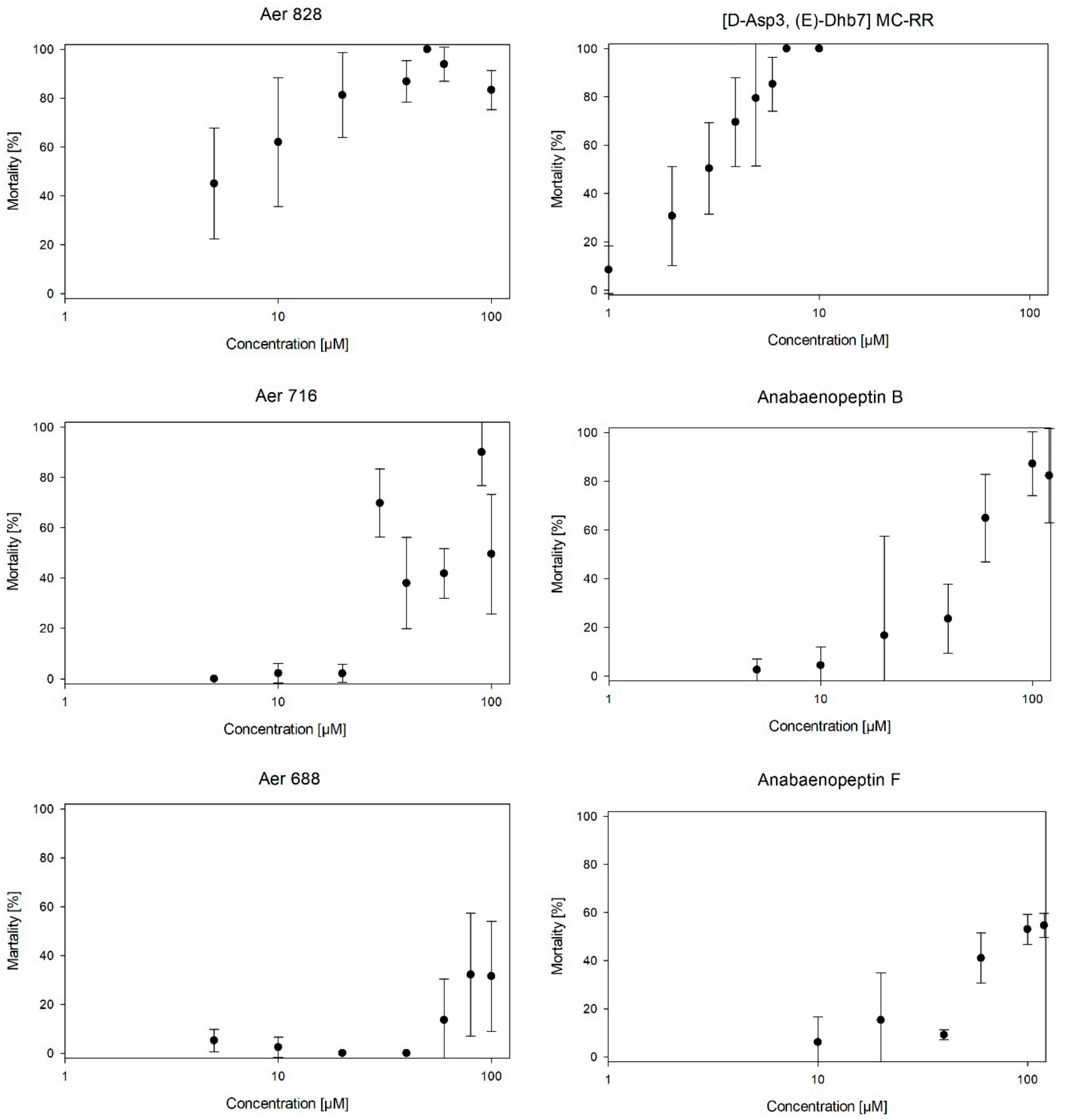

Figure S17. Percentage mortality (mean ± 1SD) through purified aeruginosin variants Aer 828A ([M+H]$^+$ 829.3), Aer 89 ([M+H]$^+$ 717.3), Aer 688 ([M+H]$^+$ 689.3) as inferred using a standard toxicity assay (i.e. *Thamnocephalus platyurus*) and compared with that of purified [D-Asp3, (E)-Dhb7] microcystin-RR ([M+H] $^+$ 1024), and anabaenopeptin B ([M+H]$^+$ 837) and anabaenopeptin F ([M+H]$^+$ 851).

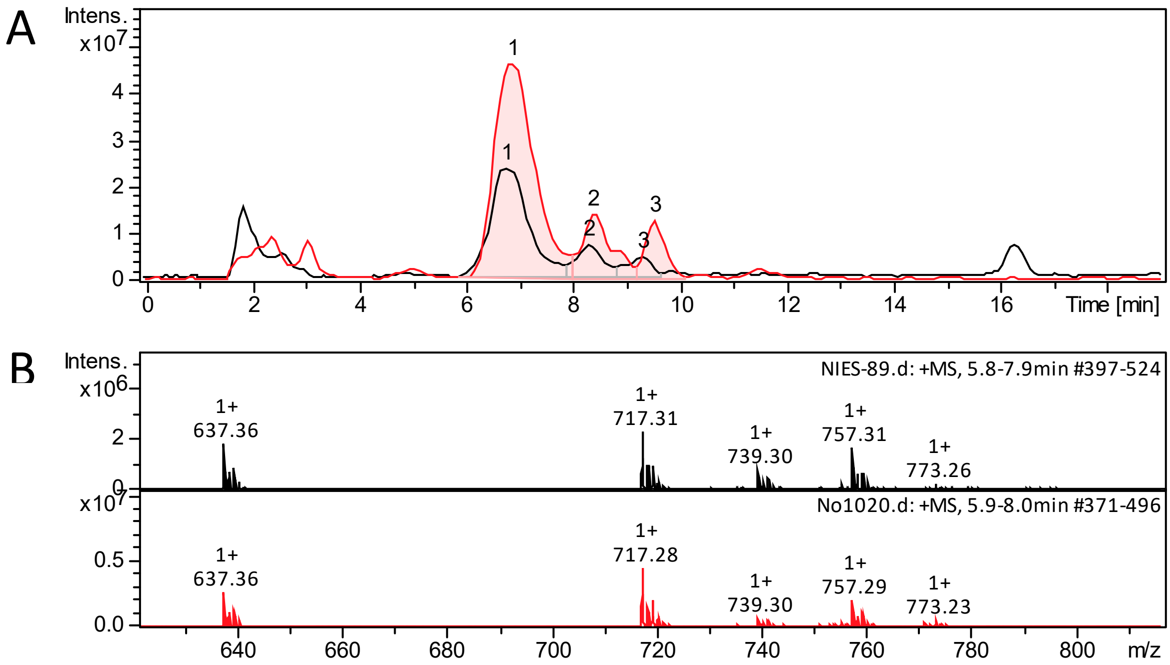

Figure S18. (A) Base peak chromatograms (BPC) of methanolic peptide extracts from *Microcystis* strain NIES-89 (black) and *Planktothrix* strain No1020 (red). **(B)** Mass peak chromatograms [m z^{-1}] of NIES-89_peak1 (black) and No1020_peak1 (red); $[M-SO_3+H]^+$ 637.3; $[M+H]^+$ 717.3; $[M+Na]^+$ 739.3; $[M+K]^+$ 757.3.

References

26. Röttig, M.; Medema, M. H.; Blin, K.; Weber, T.; Rausch, C.; Kohlbacher, O., NRPSpredictor2--a web server for predicting NRPS adenylation domain specificity. *Nucleic Acids Res* **2011,** 39, (Web Server issue), W362-7. doi: 10.1093/nar/gkr323.
